# Effect of Vitrification on the MicroRNA Transcriptome in Mouse Blastocysts

**DOI:** 10.1371/journal.pone.0123451

**Published:** 2015-04-08

**Authors:** Xueming Zhao, Haisheng Hao, Weihua Du, Huabin Zhu

**Affiliations:** Embryo Biotechnology and Reproduction Laboratory, Institute of Animal Sciences (IAS), Chinese Academy of Agricultural Sciences (CAAS), Beijing 100193, P. R. China; South China Agricultural University, CHINA

## Abstract

Vitrification is commonly used in the cryopreservation of mammalian blastocysts to overcome the temporal and spatial limitations of embryo transfer. Previous studies have shown that the implantation ability of vitrified blastocysts is impaired and that microRNAs (miRNAs) regulate the critical genes for embryo implantation. However, little information is available about the effect of vitrification on the miRNA transcriptome in blastocysts. In the present study, the miRNA transcriptomes in fresh and vitrified mouse blastocysts were analyzed by miRNA Taqman assay based method, and the results were validated using quantitative real-time PCR (qRT-PCR). Then, the differentially expressed miRNAs were assessed using the Gene Ontology (GO) and Kyoto Encyclopedia of Genes and Genomes (KEGG) databases. Overall, 760 known mouse miRNAs were detected in the vitrified and fresh mouse blastocysts. Of these, the expression levels of five miRNAs differed significantly: in the vitrified blastocysts, four miRNAs (mmu-miR-199a-5p, mmu-miR-329-3p, mmu-miR-136-5p and mmu-miR-16-1-3p) were upregulated, and one (mmu-miR-212-3p) was downregulated. The expression levels of all miRNAs measured by the miRNA Taqman assay based method and qRT-PCR were consistent. The four upregulated miRNAs were predicted to regulate 877 candidate target genes, and the downregulated miRNA was predicted to regulate 231 genes. The biological analysis further showed that the differentially expressed miRNAs mainly regulated the implantation of embryos. In conclusion, the results of our study showed that vitrification significantly altered the miRNA transcriptome in mouse blastocysts, which may decrease the implantation potential of vitrified blastocysts.

## Introduction

Blastocyst transfer has been shown to be an effective approach to improve implantation and pregnancy rates during the transfer of embryos produced in vitro [1]. To overcome the temporal and spatial limitations of embryo transfer, several types of cryopreservation methods have been developed, including controlled freezing [2] and vitrification [3]. Many experiments have shown that vitrification has a higher efficiency of cryopreservation than controlled freezing [4–7], mainly due to the higher rapid cooling rate of vitrification (>20,000°C/min) [8].

Vitrification is commonly applied in bovine [8], mouse [9], and human [10] blastocysts. However, the implantation of vitrified blastocysts is impaired. Therefore, researchers are now focusing on the possible influence of blastocyst vitrification on factors such as the inner cell mass number [11], spindle formation [12], fragmented DNA in nuclei [7,13], the expression levels of important development-related genes [14], and the sex ratio of offspring after transfer [15]. Recently, our research group reported the effect of vitrification on the promoter methylation levels and the mRNA expression levels of octamer-binding transcription factor 4 (*OCT4*), Nanog homeobox (*NANOG*), and caudal-type homeobox 2 (*CDX2*) in mouse blastocysts [16].

MicroRNAs (miRNAs) are a family of small RNAs that are 21–25 nucleotides in length and are involved in the negative regulation of gene expression at the post-transcriptional level [17,18]. Many miRNA knockout experiments have illustrated the physiological importance of individual miRNAs in mice [19–22]. Several studies have reported that miRNAs are important regulators of pluripotency and differentiation and, consequently, of early lineage segregation in embryonic development [23,24] and maternal-to-embryonic transition [25]. MiRNAs also regulate the critical genes for embryo implantation [26], and the disruption of blastocyst miRNA expression is associated with human infertility [27]. Nuclear transfer by microinjection has been found to alter the miRNA profile of enucleated mouse oocytes [28], indicating that external stimuli may affect the miRNA profiles of mammalian oocytes. Currently, however, little information is available on the effect of vitrification on the miRNA transcriptome profile in oocytes and embryos.

In the present study, the TaqMan Array Rodent MicroRNA A+B Cards Set v3.0 with an Applied Biosystems 7900 HT Fast Real-time PCR system was utilized to detect the miRNA transcriptome in fresh and vitrified mouse blastocysts. Five of 760 known mouse miRNAs were found to be differentially expressed between the vitrified and fresh mouse blastocysts. The biological analysis further showed that the differentially expressed miRNAs mainly regulated the implantation of embryos, which may partially explain the impaired implantation ability of vitrified blastocysts.

## Results

Of the 2416 blastocysts that were vitrified in this experiment, 2188 (90.56%) survived after thawing.

### miRNA expression in vitrified and fresh mouse blastocysts

**Fig 1:**
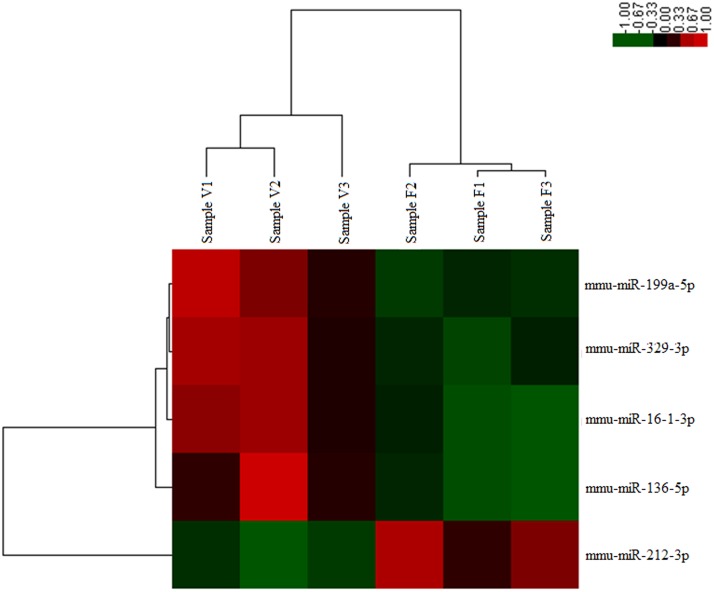
Heat map of differentially expressed miRNAs in the vitrification and control groups. F1, F2, and F3 are the samples from the fresh blastocyst group. V1, V2, and V3 are the samples from the vitrified blastocyst group. Red indicates upregulated expression, and green indicates downregulated expression, with respect to a reference expression level.

S1 Table presented the fold change of the miRNAs in vitrified blastocysts. Compared to the expression levels in the fresh blastocysts, five miRNAs showed significantly different expression in the vitrified blastocysts (Fig 1). Of these miRNAs, four were upregulated (mmu-miR-199a-5p, mmu-miR-329-3p, mmu-miR-136-5p, and mmu-miR-16-1-3p), and one was downregulated (mmu-miR-212-3p).

### Validation of miRNA array results by qRT-PCR

**Fig 2:**
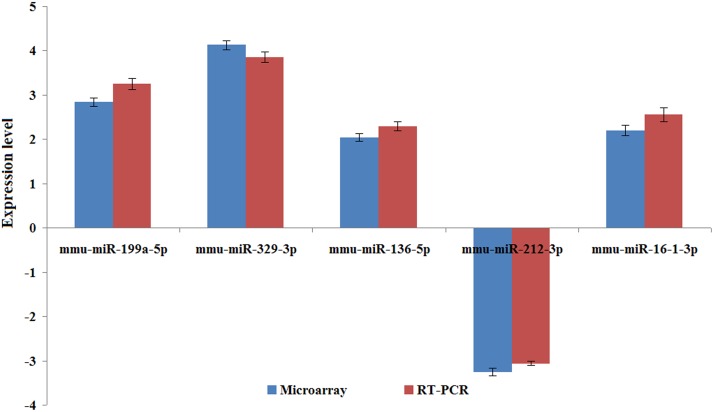
Validation of miRNA array results by qRT-PCR. Three biological replicates were used, and U6 snRNA was used as an internal control. The Y-axis denotes the fold change between the vitrified and fresh blastocyst groups.

A miRNA assay (No. 4427975) targeting the five differentially expressed miRNAs was used to confirm the results obtained from the miRNA Taqman assay based method (Fig 1). The miRNA expression levels quantified using qRT-PCR and the microarray were compared (Fig 2), and the results of both the methods were found to be consistent.

### Prediction of target genes and functional annotation

Among the upregulated miRNAs, 292, 178, 164, and 243 candidate target genes were predicted for mmu-miR-199a-5p, mmu-miR-329-3p, mmu-miR-136-5p, and mmu-miR-16-1-3p, respectively (S2 Table). For the downregulated miRNA, mmu-miR-212-3p, 231 candidate target genes were predicted (S3 Table).

GO was performed to determine the functions of the target genes regulated by these differentially expressed miRNAs. Many of the target genes were found to be involved in the regulation of gene transcription, DNA methylation, histone acetylation, and embryo implantation (Table 1 and Table 2).

**Table 1:** GO analysis of candidate target genes regulated by the upregulated miRNAs.

GO_ID	GO_name	P-value
GO:0006355	Regulation of transcription, DNA-dependent	4.33E-60
GO:0008152	Metabolic process	2.56E-09
GO:0001701	In utero embryonic development	7.14E-07
GO:0006917	Induction of apoptosis	4.05E-05
GO:0050896	Response to stimulus	0.0001535
GO:0060136	Embryonic process involved in female pregnancy	0.0002918
GO:0072593	Reactive oxygen species metabolic process	0.0007675
GO:0042771	Intrinsic apoptotic signaling pathway in response to DNA damage by p53 class mediator	0.0010413
GO:0080111	DNA demethylation	0.0010992
GO:0044027	Hypermethylation of CpG island	0.0023122
GO:0071470	Cellular response to osmotic stress	0.0023122
GO:0043407	Negative regulation of MAP kinase activity	0.0025463
GO:0010424	DNA methylation on cytosine within a CG sequence	0.0038178
GO:0032873	Negative regulation of stress-activated MAPK cascade	0.0038178
GO:0000188	Inactivation of MAPK activity	0.0038292
GO:0006309	Apoptotic DNA fragmentation	0.0038292
GO:0010629	Negative regulation of gene expression	0.0049572
GO:0006979	Response to oxidative stress	0.0052717
GO:0023019	Signal transduction involved in regulation of gene expression	0.006932
GO:0048864	Stem cell development	0.0078691

**Table 2:** GO analysis of candidate target genes regulated by the downregulated miRNA.

GO_ ID	GO_name	P-value
GO:0006355	Regulation of transcription, DNA-dependent	1.10E-27
GO:0006915	Apoptotic process	1.21E-08
GO:0009791	Post-embryonic development	4.24E-06
GO:0043066	Negative regulation of apoptotic process	5.24E-05
GO:0006349	Regulation of gene expression by genetic imprinting	8.11E-05
GO:0010468	Regulation of gene expression	0.0001458
GO:0035067	Negative regulation of histone acetylation	0.0011141
GO:2000036	Regulation of stem cell maintenance	0.0011141
GO:0016573	Histone acetylation	0.0062976
GO:0032259	Methylation	0.0069064
GO:0034721	Histone H3-K4 demethylation, trimethyl-H3-K4-specific	0.0079573
GO:0070512	Positive regulation of histone H4-K20 methylation	0.0079573
GO:2000617	Positive regulation of histone H3-K9 acetylation	0.0079573
GO:2000620	Positive regulation of histone H4-K16 acetylation	0.0079573
GO:0016571	Histone methylation	0.008903

KEGG pathway annotations of the candidate target genes regulated by differentially expressed miRNAs were also performed. A number of KEGG pathways were identified as relevant to embryo implantation (Table 3 and Table 4).

**Table 3:** Significant pathways of candidate target genes regulated by the upregulated miRNAs.

Path_ID	Path_name	Path_gene_count	P-value
4310	Wnt signaling pathway	148	8.22E-12
4510	Focal adhesion	205	3.28E-11
4520	Adherens junction	75	1.24E-09
4910	Insulin signaling pathway	141	2.85E-08
4010	MAPK signaling pathway	259	7.37E-08
4530	Tight junction	138	8.83E-06
4115	p53 signaling pathway	69	0.0001053
4150	mTOR signaling pathway	62	0.0004703
4630	Jak-STAT signaling pathway	156	0.0007667

**Table 4:** Significant pathways of candidate target genes regulated by the downregulated miRNA.

Path_ID	Path_name	Path_gene_count	P-value
4010	MAPK signaling pathway	259	2.17E-07
4310	Wnt signaling pathway	148	3.25E-07
4510	Focal adhesion	205	3.82E-06
4915	Estrogen signaling pathway	99	0.000100728
4370	VEGF signaling pathway	66	0.000289995
4520	Adherens junction	75	0.000475568
4910	Insulin signaling pathway	141	0.00053183
4150	mTOR signaling pathway	62	0.003954979
4530	Tight junction	138	0.004656746

### Validation of changes in mRNA expression of target genes

**Fig 3:**
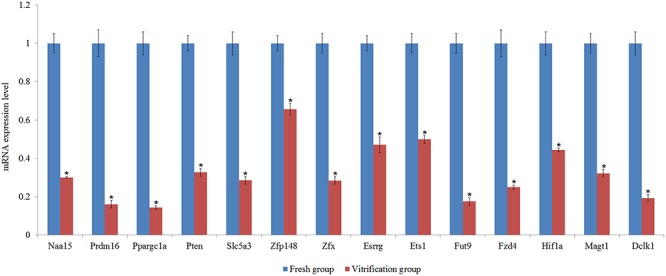
Validation of changes in mRNA expression of target genes. Means of vitrification group with star on the columns differed significantly with those of the fresh group (P<0.05).

From candidate target genes of these differentially expressed miRNAs, 14 genes were randomly selected to quantify their mRNA expression levels (S4 Table). The results showed that vitrification significantly altered the mRNA expression level of all these selected genes (Fig 3).

## Discussion

In this study, 5 of the 760 identified miRNAs, namely, mmu-miR-199a-5p, mmu-miR-329-3p, mmu-miR-136-5p, mmu-miR-16-1-3p, and mmu-miR-212-3p, showed significantly different expression between the vitrified and fresh mouse blastocysts.

### Functions of the differentially expressed miRNAs

Of the five differentially expressed miRNAs, four miRNAs, namely, mmu-miR-199a-5p, mmu-miR-329-3p, mmu-miR-136-5p, and mmu-miR-16-1-3p, were significantly upregulated in the vitrified blastocysts.

The mmu-miR-199a-5p miRNA showed the highest expression level at the embryonic stage at 12.5 days postcoitus [29]. A previous study reported that mmu-miR-199a-5p could silence the self-renewal of mouse embryonic stem cells [30]. Mmu-miR-199a-5p is upregulated during the fibrogenic response to tissue injury and mediates TGFbeta-induced lung fibroblast activation by targeting caveolin-1 [31]. Mmu-miR-199a* was found to be differentially expressed in the mouse uterus during implantation in coordination with the expression of cyclooxygenase-2 (*COX-2*), a gene critical for implantation [26]. In humans, miR-329 may inhibit cell proliferation in human glioma cells by regulating E2F1-mediated suppression of the Akt pathway [32]. Mmu-mir-16 is reported to be related to apoptosis in mouse embryos [33], and miRNA profiling suggested that genes targeted by this miRNA are important in spindle organization in mouse oocytes [34]. In *Drosophila*, miR-316 is expressed in the mesoderm [35].

In our study, mmu-miR-212-3p was downregulated in vitrified mouse blastocysts. Previously, mmu-miR-212 was found to post-transcriptionally regulate C-terminal-binding protein 1 [36], a protein that was recently shown to repress the expression of steroidogenic factor 1 [37], a nuclear receptor involved in ovarian, adrenal, and testis development and function [38]. In mouse periovulatory granulosa cells, mmu-miR-212 was found to be highly upregulated following luteinizing hormone (LH)/hCG induction [36].

Thus, the miRNAs that were found to be differentially expressed in the vitrified blastocysts are mainly involved in embryo implantation and cell development.

### GO analysis of candidate genes regulated by differentially expressed miRNAs in vitrified blastocysts

The GO analysis of the candidate target genes regulated by the differentially expressed miRNAs illustrated a high enrichment of genes involved in the regulation of transcription, implantation, metabolism, DNA methylation, and histone acetylation.

The results of the present study indicated that the candidate target genes regulated by significantly altered miRNAs induced by vitrification (such as GO:0006355, GO:0010629, GO:0006349, and GO:0010468) are involved in the regulation of transcription, which may partially explain the mechanisms through which vitrification alters the expression levels of pluripotent genes in mouse blastocysts [16], development- [14] and stress-related [39] genes in bovine blastocysts and the gene expression profiles of in vivo-derived 8-cell stage mouse embryos [40].

Previous experiments have reported that rabbit embryo vitrification reduced early fetal growth [41], increased losses throughout gestation [41,42], and affected gene and protein expression in the placenta at day 14 after the transfer of vitrified morulae [43]. A higher rate of major postpartum hemorrhage was observed in humans receiving vitrified blastocyst transfers [10]. The present study showed that the candidate target genes of miRNAs whose expression levels were significantly altered by vitrification (e.g., GO:0001701, GO:0060136, and GO:0009791) were involved in the regulation of implantation; these results explained the impaired implantation ability of vitrified blastocysts.

The target genes regulated by miRNAs whose expression levels were significantly altered by vitrification (e.g., GO: 0008152 and GO: 0072593) are also involved in the regulation of metabolism: this may explain the altered energy metabolism reported in vitrified blastocysts [44] and oocytes [45–47], as well as the altered oxygen metabolism in blastocysts [44] and oocytes [45,46].

In oocytes, vitrification has been reported to significantly alter the overall methylation levels in the *OCT4* and sex determining region y-box 2 (*SOX2*) promoters in mice [48] and the H4 acetylation and H3K9 methylation levels in mice [49] and pigs [50]. Moreover, vitrification has been shown to significantly alter the imprinted cortical region methylation of *H19* in bovine embryos [51], the *H19* differentially methylated domain in mouse fetuses [52], and the methylation levels of the *OCT4*, *NANOG*, and *CDX2* promoters in mouse blastocysts [16]. The results of the present study indicated that candidate target genes regulated by miRNAs whose expression levels were significantly altered by vitrification (e.g., GO:0080111, GO:0044027, GO:0016573, and GO:0032259) are involved in the regulation of methylation and acetylation; these findings may explain the altered methylation and acetylation levels in vitrified embryos or oocytes.

### Pathway analysis of candidate genes regulated by the differentially expressed miRNAs

The results of the present study indicated that the significant pathways of the candidate target genes are mainly involved in embryo implantation, such as the Wnt, adherens, and tight junction genes (Table 3 and Table 4), which partially explains the reduced implantation ability and post-implantation development potential of vitrified embryos [41,42].

Wnt signaling plays an important role in mouse embryonic development and is involved in processes such as axis patterning, cell fate specification, cell proliferation, and cell migration [53], as well as in the coordination of mouse uterus—embryo interactions required for implantation [54]. Both adherens and tight junction proteins are tightly regulated at implantation sites [55]; therefore, trophectodermal cells must become mobile and penetrate between the endometrial luminal epithelial cells [56]. Focal adhesions play an important role in promoting embryo invasion; in particular, they disassemble at the time of implantation in rats, facilitating the detachment of the uterine luminal epithelium to allow the embryo to invade the endometrium [57]. The insulin signaling pathway is involved in the pregnancy of murine blastocysts [58]. The Jak/STAT pathway is activated by the actions of multiple cytokines such as IL11 and LIF, which are known for their roles in implantation [59]. VEGF can act on human blastocysts, enhancing their outgrowth and adhesive capacity [60]. mTOR signaling leads to the development of mouse trophoblast cell motility and the initiation of implantation [61]. Maternal estrogen signaling induces adhesion complex assembly in the trophectoderm [62]. The MAP kinase pathway has been implicated in stem cells [63].

### Validation of changes in mRNA expression of target genes

In the present study, the mRNA expression levels of these selected genes regulated by the differentially expressed miRNAs were all decreased by the vitrification. It has been reported that the predominant regulatory effect of miRNAs is to repress their target mRNAs; mechanisms for this include translational repression, mRNA cleavage, mRNA deadenylation or alteration of mRNA stability [64], which contributes to explain the mRNA expression changes of these selected genes in the present study.

## Conclusions

By using the TaqMan Array Rodent MicroRNA A+B Cards Set v3.0 with an Applied Biosystems 7900 HT Fast Real-time PCR system, the expression levels of 760 known miRNAs were compared between fresh and vitrified mouse blastocysts, and five of these mRNAs were found to differ significantly between the two blastocyst groups. The expression levels of all miRNAs measured by the miRNA Taqman assay based method and qRT-PCR were consistent. Bioinformatic analysis of the differentially expressed miRNAs showed that major affected pathways are known to be involved in implantation [41,42], which partially explain the impaired implantation ability of vitrified blastocysts.

## Materials and Methods

### Ethics Statement

All animal procedures were performed according to the guidelines developed by the China Council on Animal Care, and all protocols were approved by the Animal Care and Use Committee of Beijing, China. The approval ID or permit numbers were *SYXK (Beijing) 2008–007* and *SYXK (Beijing) 2008–008*.

### Chemicals

All chemicals and media were purchased from Sigma Chemical Co. (St. Louis, MO, USA), and all plasticware was obtained from Nunc-ware (Nunc; Nalge Nunc International, Roskilde, Denmark), unless indicated otherwise.

### Open-pulled straw (OPS) preparation

OPSs were prepared according to the method described previously [8]. Briefly, 0.25-mL plastic straws (IMV, L’Aigle, France) were heat-softened and manually pulled to a length of 2–3 cm to attain an approximate outer diameter of 0.23 mm and a wall thickness of 0.02 mm.

### Vitrification solutions

The pretreatment solution consisted of 10% ethylene glycol (EG) and 10% dimethyl sulfoxide (DMSO) in Dulbecco’s phosphate-buffered saline (DPBS) containing 3 mg/mL bovine serum albumin (BSA). EDFS30 solution, which was used for vitrification, contained 15% EG and 15% DMSO in Ficoll-sucrose (FS) solution (DPBS containing 300 mg/mL Ficoll, 171.2 g/L sucrose, and 3 mg/mL BSA).

### Collection of blastocysts

Five week-old Kunming white mice were superovulated with an intraperitoneal (ip) injection of 10 IU equine chorionic gonadotropin (eCG; Ningbo Hormone Products Co., Ltd, Zhejiang, China) followed by an ip injection of 10 IU human chorionic gonadotropin (hCG; Ningbo Hormone Products Co., Ltd.) 48 hours later. The mice were then mated with fertile males, and the successfully mated females were identified the next day by detecting a vaginal plug. Blastocysts were collected from the excised oviducts of mated females within 92–94 hours after the hCG injection.

### Vitrification and thawing of blastocysts

The embryos were equilibrated in pretreatment solution for 30 seconds, loaded into an OPS with EDFS30 for 25 seconds, and plunged into liquid nitrogen (LN_2_).

The blastocysts were thawed by rinsing in 0.5 M sucrose under mineral oil for 5 minutes and washed three times in CZB medium.

Blastocysts with morphologically intact inner cell mass, trophoderm, and re-expanding blastocoele at 18–20 hours after warming were considered to have survived, according to the method described by Son et al. [65].

### Total RNA isolation

Total RNA was extracted from each pool of 300 (n = 3) vitrified/fresh blastocysts using an RNeasy Mini Kit (Qiagen, CA, USA) according to the manufacturer’s instructions. Genomic DNA contamination was removed by treating the isolated RNA with DNase using a DNA-free kit (Ambion, TX, USA). TriPure Isolation Reagent (Roche, NSW, Australia) was used to purify the extracted RNA. A Nanodrop spectrophotometer (Thermo-Fisher) was utilized to measure the concentrations of total RNA.

### MiRNA array

MiRNAs were measured in triplicate using the TaqMan Array Rodent MicroRNA A+B Cards Set v3.0 with an Applied Biosystems 7900 HT Fast Real-time PCR system according to the method described by Granjon et al. [66].

Reverse transcription of 30 ng of total RNA from fresh and vitrified blastocysts was carried out using a TaqMan MicroRNA Reverse Transcription Kit. TaqMan PreAmp Master Mix was used to pre-amplify small amounts of cDNA without introducing amplification bias to the sample. The pre-amplified products were diluted in the ratio of 1:5 and then used as a template for the PCR reaction and loaded into the corresponding fill port. Individual singleplex PCR reactions were carried out in 384-well plates, and the miRNA expression level was measured using Ct (threshold cycle) determined by RQ Manager software. U6 snRNA was used as an endogenous control to normalize the data, as described in previous studies [67–69]. Microarray data have been deposited in the NCBI’s Gene Expression Omnibus database (accession number GSE62581).

The Ct value for each miRNA was calculated using an ABI 7900HT Fast Real-time PCR system. Raw Ct values considered “undetermined” by the software or at a level of ≥40 cycles were excluded from the analysis. Relative quantification was calculated using the comparative Ct (2^–ΔΔCt^) method [70].

The qRT-PCR data were analyzed by Student's *t*-test and calculated using Statistical Analysis System (SAS) software (SAS Institute; Cary, NC, USA). Each experiment was repeated three times, and a P-value of <. 05 was considered statistically significant.

### Validation of miRNA array results by qRT-PCR

The MiRNA assay (ABI) was used to confirm the results of the miRNA Taqman assay based method. qRT-PCR was performed using a TaqMan Universal PCR Master Mix II and 7500 Fast system (Applied Biosystems, CA, USA) with the comparative Ct (2^–ΔΔCt^) method [70]. The isolation and reverse transcription of total RNA and preamplification of cDNA were performed as described previously.

### Gene target prediction and functional annotation

MiRanda (http://www.microrna.org/microrna/) and TargetScan (http://www.targetscan.org/) were utilized to scan targets for the differentially expressed miRNAs obtained above. The intersection of these two prediction programs was selected in the present study. The thresholds of the candidate target sites were an exact match to positions 2–7 of the mature miRNA with a downstream ‘A’ across from position 1 of the miRNA, to positions 2–8 of the mature miRNA, or to positions 2–8 of the mature miRNA with a downstream ‘A’ across from position 1 of the miRNA. The 3’-untranslated region (UTR) sequences of the target genes in mice were obtained from the National Center for Biotechnology Information (NCBI) database.

The main functions of the candidate target genes regulated by differentially expressed miRNAs were determined using the Gene Ontology (GO) analysis. GO analysis, which was provided by the NCBI, was used to organize genes into hierarchical categories and uncover the gene regulatory network on the basis of biological processes and molecular functions [71]. Significance was set at P <. 01.

The Kyoto Encyclopedia of Genes and Genomes (KEGG) database (http://www.genome.jp/kegg/) was used to perform pathway annotations of the candidate target genes regulated by differentially expressed miRNAs. Significance was set at P <. 01.

### Validation of changes in mRNA expression of target genes

Total RNA was isolated from each pool (n = 3) containing 100 fresh or vitrified blastocysts according to the method as mentioned above and dissolved in sterile water at -80°C until use. First-strand cDNA was synthesized using 0.05 μg RNA with random hexamers. qRT-PCR was performed using an ABI 7500 Fast system with the comparative Ct (2^–ΔΔCt^) method [70]. The β-actin gene was used as a reference gene, and the primers for each gene were listed in S4 Table.

## Supporting Information

S1 Table Fold change in the expression of miRNAs in vitrified blastocysts. (XLS)

S2 Table Candidate target genes regulated by the upregulated miRNAs (mmu-miR-199a-5p, mmu-miR-329-3p, mmu-miR-136-5p and mmu-miR-16-1-3p). (XLS)

S3 Table Candidate target genes regulated by the downregulated miRNA (mmu-miR-212-3). (XLS)

S4 Table Primers used for the validation of changes in mRNA expression of target genes. (DOCX)
