# Supplementary material for: Effect of Vitrification on the MicroRNA Transcriptome in Mouse Blastocysts
Source: PLoS One. 2015 Apr 8;10(4):e0123451. doi: 10.1371/journal.pone.0123451 (PMC4390370; doi:10.1371/journal.pone.0123451)
Supplement: S4 Table — (DOCX) [file pone.0123451.s004.docx]

**Table S4. Primers used for the validation of changes in mRNA expression of target genes**

| Differentially  expressed miRNAs | Target genes | Gene ID | Primer sequence (5` to 3`) |
| --- | --- | --- | --- |
| mmu-miR-199a-5p, mmu-miR-329-3p | Naa15 | 74838 | F: CGATTCCGATGCCACCC  R: AATTAACTGAATATCAACGGGGTAAA |
| mmu-miR-199a-5p, mmu-miR-136-5p | Prdm16 | 70673 | F: TTTTTGGCATTGGACAACTTATTC  R: GGCAGTGGTGACCGTGGAA |
| mmu-miR-199a-5p, mmu-miR-136-5p, mmu-miR-16-1-3p | Ppargc1a | 19017 | F: ATGGCACGCAGCCCTATTC  R: ACCTGGAATATGGTGATCGGG |
| mmu-miR-329-3p, mmu-miR-16-1-3p | Pten | 19211 | F: TTTGAAGCGTGGTGCGTGA  R: GGGGCAAGGTAGGTACGCAT |
| mmu-miR-329-3p, mmu-miR-16-1-3p | Slc5a3 | 53881 | F: AATGATTTCCAGGATAGTGTTTGCT  R: CCACAGGAACCAAGGTCATCAC |
| mmu-miR-199a-5p, mmu-miR-16-1-3p | Zfp148 | 22661 | F: CCACCAGGCTTACCAAATGAG  R: CTTGTCCCTGGTCCCCGA |
| mmu-miR-329-3p, mmu-miR-16-1-3p | Zfx | 22764 | F: ATCCTTACCAAGTCTTTCACAGTTTT  R: GTGATGTGCCAGACCCGTGT |
| mmu-miR-329-3p, mmu-miR-136-5p,  mmu-miR-16-1-3p | Esrrg | 26381 | F: TGTGCTTAGTGTGTGGCGACA  R: ACTCTATGTTACCTTGAATCGTCCTC |
| mmu-miR-199a-5p, mmu-miR-16-1-3p | Ets1 | 23871 | F: TTGGACTTAGTGTTAGTTACGGGG  R: TTGAACTGTGCTATGATGCCGA |
| mmu-miR-199a-5p, mmu-miR-16-1-3p | Fut9 | 14348 | F: ATAGCACCGTGGAGTAGTTCAGC  R: AGACGATTAGAAATGGGCGAAG |
| mmu-miR-199a-5p, mmu-miR-136-5p | Fzd4 | 14366 | F: AACTGCTGGAGAGGCGACTG  R: GGCTGATCTAGCAGACACCCC |
| mmu-miR-199a-5p, mmu-miR-16-1-3p | Hif1a | 15251 | F: GAATCAAAAACAGAGACGAAGGAC  R: CTGATGCCTTAGCAGTGGTCGT |
| mmu-miR-199a-5p, mmu-miR-16-1-3p | Magt1 | 67075 | F: GCAGGAGGCTTGGAGTGGA  R: CGGCTTATGTGATGTTGTGGTT |
| mmu-miR-329-3p, mmu-miR-136-5p | Dclk1-A | 13175 | F: CAGCCTAACCCTAGAGATTGACAGT  R: TGGTTCCCCGAGGGTCTG |
| _____ | β-actin | 11461 | F: CGTTGACATCCGTAAAGACCTC  R: ACATCTGCTGGAAGGTGGACA |
